# Biochemical and structural characterization of a glucan synthase *GFGLS2* from edible fungus *Grifola frondosa* to synthesize β-1, 3-glucan

**DOI:** 10.1186/s13068-023-02380-6

**Published:** 2023-10-30

**Authors:** Yu-Meng Yang, Xin Fu, Feng-Jie Cui, Lei Sun, Xin-Yi Zan, Wen-Jing Sun

**Affiliations:** 1 School of Food and Biological Engineering, Jiangsu University, Zhenjiang, 212013 China; 2 Jiangxi Provincial Engineering and Technology Center for Food Additives Bio-Production, Dexing, 334221 China

**Keywords:** *Grifola frondosa*, β-1, 3-Glucan synthase, Glucan synthesis, AlphaFold, Catalytic mechanism

## Abstract

**Background:**

*Grifola frondosa* is a Basidiomycete fungus belonging to the family of Grifolaceae and the order of Polyporales. β-Glucans are the main polymers in *G. frondosa*, playing a crucial role in the physiology and representing the healthy benefits for humans. The membrane-integrated β-1, 3-glucan synthase (GLS) is responsible for glucan synthesis, cell wall assembly, differentiation and growth of the edible fungi. However, the structural/catalytic characteristics and mechanisms of β-1, 3-glucan synthases in *G. frondosa* are still unknown due to their extremely complex structures with multi-transmembranes and large molecular masses.

**Results:**

Herein, a β-1, 3-glucan synthase (GFGLS2) was purified and identified from the cultured mycelia with a specific activity of 60.01 pmol min^−1^ μg^−1^ for the first time. The GFGLS2 showed a strict specificity to UDP-glucose with a V_max_ value of 1.29 ± 0.04 µM min^−1^ at pH 7.0 and synthesized β-1, 3-glucan with a maximum degree of polymerization (DP) of 62. Sequence Similarity Network (SSN) analysis revealed that GFGLS2 has a close relationship with others in *Ganoderma sinense*, *Trametes coccinea*, *Polyporus brumalis*, and *Trametes pubescens*. With the assistance of 3D structure modelling by AlphaFold 2, molecular docking and molecular dynamics simulations, the central hydrophilic domain (Class III) in GFGLS2 was the main active sites through binding the substrate UDP–glucose to 11 amino acid residues via hydrogen bonds, π-stacking and salt bridges.

**Conclusions:**

The biochemical, 3D structural characterization and potential catalytic mechanism of a membrane-bound β-1, 3-glucan synthase GFGLS2 from cultured mycelia of *G. frondosa* were well investigated and would provide a reasonable full picture of β-1, 3-glucan synthesis in fungi.

## Background

Edible fungi are generally regarded a safe and low-calorie food and nutritious dietary supplement in many countries including China, USA and Japan [1]. The richness of polysaccharides, proteins and vitamins contribute to their various functions including antioxidant, anticancer, immunoregulatory and anti-aging activities [2]. α/β-Glucans, containing 1, 3-, 1, 4- and/or 1, 6-glucosidic linkages, are recognized as the main contributors to various nutritional and supplementary credits of edible fungi, and have been intensively investigated to evaluate their potencies for industrial application. For example, Patra et al. obtained a water soluble polysaccharide (PGPS) containing α-1, 3, and α-1, 4-Glc moieties from edible fungus *Polyporus grammocephalus* with the potential ability to stimulate the immune system components including macrophages, splenocytes, and thymocytes [3]. As summarized from some fungal species such as *Saccharomyces cerevisiae*, *Aspergillus fumigatus*, and *Neurospora crassa*, the synthase complex (GSC), consisting of a catalytic subunit glucan synthase (GLS or FKS) and at least one regulatory subunit Rho-type small GTPase, is responsible for β-1, 3-glucan synthesis [4, 5]. The synthesis process takes place at the periplasmic space to form the cross-linked polysaccharide complex by transglycosylases anchored to the plasma membrane or the cell wall [6]. Similar to other kinds of fungi, the most abundant α/β-1, 3- glucans in edible fungi probably exist in the cell walls to balance the osmotic pressure and/or other environmental stresses [7]. However, the precise locations, synthesis spaces, synthesis pathway and associated enzymes of α/β‐glucans in edible fungi are still lacking the convincing answers.

Since the first cloning a* S. cerevisiae* glucan synthase gene in 1994, GLS genes in *Candida albicans*, *Cryptococcus neoformans*, and *Paracoccidiodes brasiliensis* are proved to be conserved in fungi [8, 9]. Majority of β-1, 3-fungal glucan synthases had the large molecular masses ranging from 180 to 280 kDa, with over 10 multi-transmembrane helices (TMHs) [10]. The TMHs are clustered in two regions separated by a central hydrophilic loop facing the cytoplasmic side [11]. For example, Inoue et al. proved the β-1, 3-glucan synthase of *S. cerevisiae* containing up to 16 TMHs with a molecular mass of 200-kDa [12]. Our previous results also indicated that β-1, 3-glucan synthase CMGLSp of *Cordyceps militaris* has 15 TMHs with the predicted molecular masses of 221.7 kDa [10]. However, the functions and the catalytic characteristics/mechanisms of β-1, 3-glucan synthases in fungi are still bottlenecked by their extremely complex structures with multi-transmembranes and large molecular masses. The conserved motif QXXRW or R/KXGG in some glucan synthase has been regarded as the key motif responsible for the binding substrate UDP–glucose for polymerization of glucan chain, while no similar motif was observed in the central hydrophilic catalytic domain in β-1, 3-glucan synthase CMGLSp of *C. militaris* [10]. Hence, the fine architectures and the molecular insights on the catalytic mechanism of fungal β-1, 3-glucan synthases need to be comprehensively elucidated.

*Grifola frondosa* is a well-known highly valued edible/medicinal fungus and contains various polysaccharides including α/β-glucans and heteropolysaccharides showing immunoregulating, antitumor, hypoglycemic and hypolipidemic activities [13]. For instance, D-Fraction from *G. frondosa* fruiting bodies has exerted its antitumor effect in tumor-bearing mice by enhancing the immune system through activation of macrophages, T cells and natural killer (NK) cells [14]. Our group had made some attempts to reveal the roles of polysaccharide synthesis-associated enzymes on mycelial growth and polysaccharide/glucan synthesis in *G. frondosa.* The most important advances included the correction and re-assembly of the full sequence of two β-1, 3-glucan synthase genes *GFGLS* and *GFGLS2* in *G. frondosa* with full sequences of 5, 927 bp and 5, 944 bp (GenBank: MK80801.9 and MN477285.1) for the first time [5, 15]. GFGLS2p and GFGLSp were predicted as the 203.66 kDa and 195.2 kDa membrane-bound proteins with a high protein similarity of 72.56%, and contained two large membrane domains of 6 transmembrane helices (TMHs) at the N-terminus and 9 TMHs at the C-terminus [5, 15]. Using dual-promoter RNA silencing vectors to downregulate *GFGLS*, *GFGLS2* and *GFGLS/GFGLS2* expression, *GFGLS2* was found to play major roles in mycelial growth and polysaccharide synthesis of *G. frondosa* [5]. However, the catalytic characteristics and structural basis of glucan synthases in *G. frondosa* remains enigmatic. Hence, on the basis of our previous results, the present study aimed to predict the precise three-dimensional (3D) conformation with AlphaFold modeling, characterize their catalytic features using product entrapment purification and heterologously expressed the hydrophilic domains in GFGLS, and explain the potential catalytic mechanism by determining the synthesized products and using molecular docking, molecular dynamics simulation (DSF). The findings of the present study would provide a molecular insight on fine architectures and catalytic mechanism of β-1, 3-glucan synthases in the edible fungus *G. frondosa*.

## Results

### Purification and identification of membrane-bound β-1, 3-glucan synthase GFGLS2p

Over 175 mg of the membrane pellets yielded from 300 g of *G. frondosa* mycelia with a specific activity of 0.72 pmol min^−1^ μg^−1^. Approximate 85 mg of crude β-1, 3-glucan synthase with a specific activity of 22.74 pmol min^−1^ μg^−1^ was recovered after product entrapment purification, and 0.14 mg of Superdex 200-purified β-1, 3-glucan synthase was finally obtained with an increased specific activity of 60.01 pmol min^−1^ μg^−1^ with a purification fold of 83.34.

**Fig. 1:**
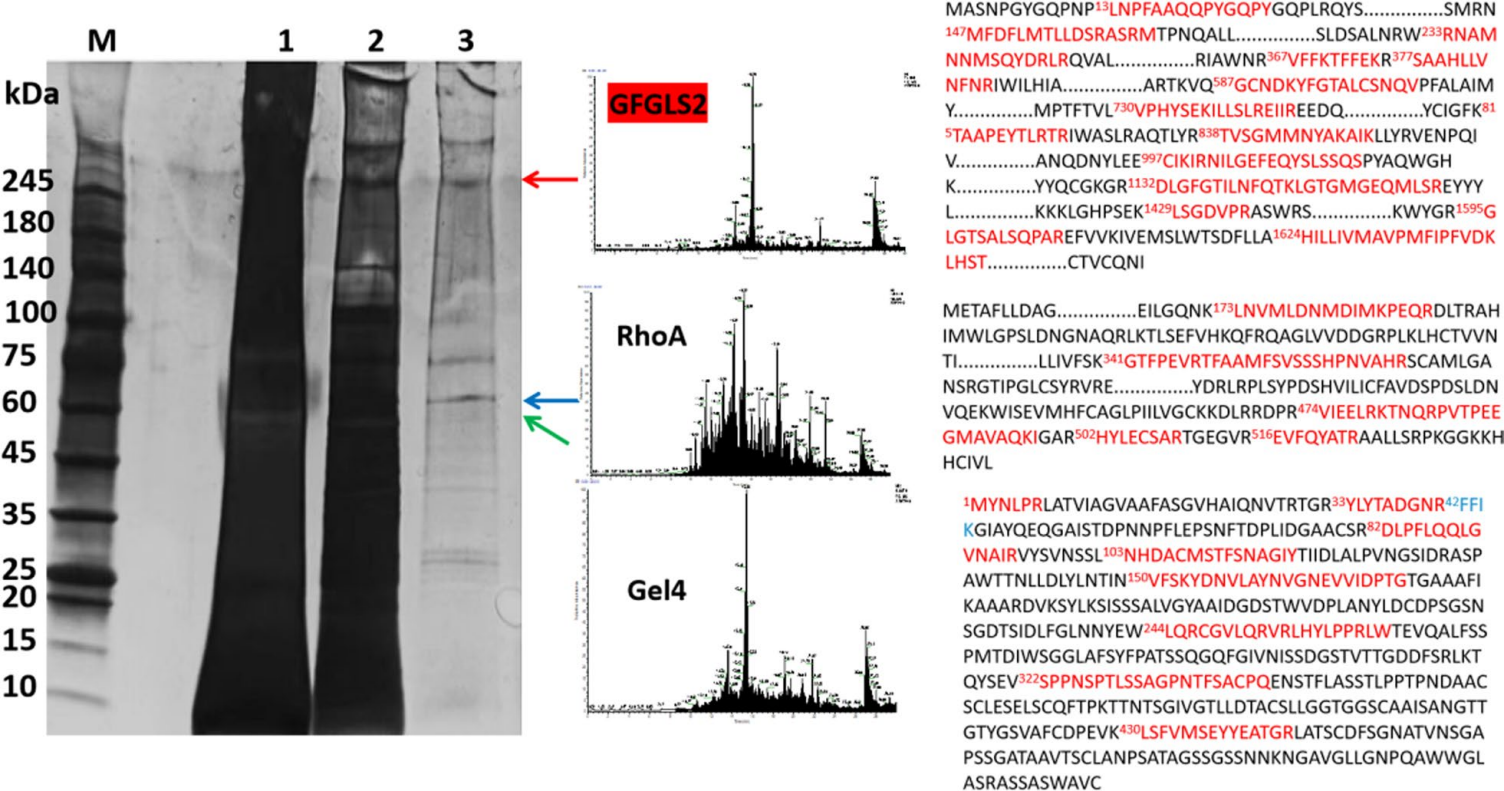
Electrophoresis analysis and mass spectrometry identification of the purified β-1, 3-glucan synthase in *G. frondosa* using Superdex 200 Increase 10/300 GL method. Lane M: maker; lanes 1–2: affinity-purified crude protein of GFGLS2; and lane 3: purified GFGLS2. Three bands of purified GFGLS2 of *G. frondosa* on the SDS-PAGE were digested with trypsin following LC–MS/MS and MaxQuant search analysis

As shown in Fig. 1, at least 8 protein bands were observed on the SDS–PAGE of partially-purified β-1, 3-glucan synthase sample (Lane 3). After trypsin digestion following LC–MS/MS and Mascot search analysis, fourteen peptides of the protein band migrating at approximately 200 kDa were matched to a β-1, 3-glucan synthase GFGLS2 (accession number: QIR82081, MW195.2 kDa) in *G. frondosa*, which confirmed that GFGLS2 is the predominate β-1, 3-glucan synthase and plays major roles in mycelial growth and polysaccharide synthesis [5]. Nine peptides of the protein band at 54 kDa were matched to a glycosylphosphatidylinositol (GPI)-anchored protein, 1, 3-beta-glucanosyltransferase gel4 (accession number: OBZ79073, MW 54.01 kDa). Different from our previous results [10], RhoA (accession number: OBZ71711, MW 59.37 kDa) was matched to the protein band at 60 kDa, which showed a similar function of Rho1 as a GTP-binding protein and a predominant regulatory component regulating β-1, 3-glucan synthase activity.

### Recombinant expression of hydrophilic cytoplasmic domains rGFGLS2-classI and rGFGLS2-classIII

**Fig. 2:**
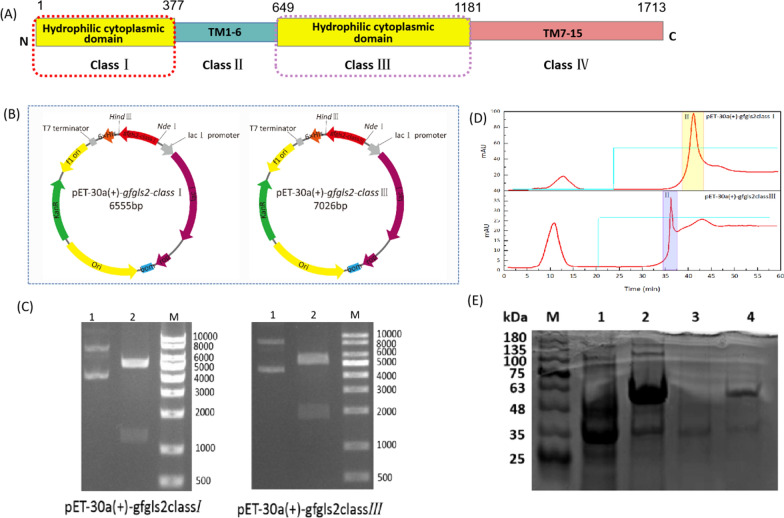
Plasmid construction process (**A**, **B**). The plasmid pET-30a ( +)-*gfgls2classI/III* was constructed by inserting the *gfgls2classI/III* gene into the *NdeI* and *HindIII* sites of plasmid pET-30a ( +). **C** The electrophoretogram of recombinant plasmids and the recombinant plasmid double-digestion product. Lane M: DNA 10,000 maker; lane 1: recombinant plasmid; and lane 2: recombinant plasmid double-digested by *NdeI* and *HindIII*. **D** Affinity purification of rGFGLS2-classI and rGFGLS2-classIII on a Ni–NTA-Sefinose column. Peak I represents the specific activity of miscellaneous proteins and peak II the imidazole-eluted target protein rGFGLS2-classI and rGFGLS2-classIII. **E** SDS-PAGE of purified pET-30a ( +)-*gfgls2classI/III*. Lane M: protein marker; lane 1 and 2: affinity-purified crude protein of pET-30a ( +)-*gfgls2classI* and pET-30a ( +)-*gfgls2classIII* loaded on the Ni–NTA-Sefinose column; and lane 3 and 4: purified pET-30a ( +)-*gfgls2classI* and pET-30a ( +)-*gfgls2classIII*

The predicted topology of GFGLS2p structure indicated that GFGLS2p contains two hydrophilic cytoplasmic domains (class I: aa 1–377 and class III: aa 649–1181). After codon-optimization, expression plasmid and strain construction, the band with an Mw of approximately ~ 43 kDa and ~ 60 kDa matching the expected molecular mass of rGFGLS2-classI and rGFGLS2-classIII were observed in the cell lysis solution of recombinant strains expression strains *E. coli* BL21(DE3)/pET-30a ( +)-*gfgls2classI* and *E. coli* BL21(DE3)/pET-30a ( +)-*gfgls2classIII*, respectively (Fig. 2A–C). The rGFGLS2-classI and rGFGLS2-classIII with high purity were obtained by ultrasonic extraction, re-solubilization and affinity chromatography, showing a symmetrical elution peak (II) and a band with a predicted M_W_ of ~ 43 kDa and ~ 60 kDa on SDS–PAGE, respectively (Fig. 2D, E).

### Substrate specificity

**Fig. 3:**
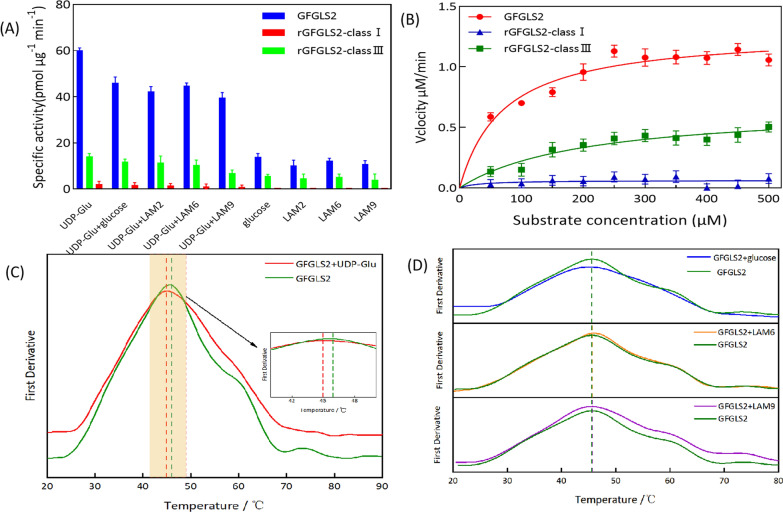
Characterizing GFGLS2 protein thermal stability by using Differential scanning fluorescence (DSF). Mean values (N = 3) were used to construct the curves. **A** Substrate specificity of β-1, 3-glucan synthase GFGLS2 and rGFGLS2-classI and rGFGLS2-classIII. **B** Kinetic parameters of the GFGLS2, rGFGLS2-classI and rGFGLS2-classIII. **C** DSF value of GFGLS2 and the UDP-Glu ligand. **D** To determine the effect of different sugars as substrate on GFGLS2 structure, Tm was tested in the presence of sugars including UDP-Glu, glucose, LAM6, LAM9

Accordingly, the substrate specificity of GFGLS2, rGFGLS2-classI and rGFGLS2-classIII were evaluated using UDP–glucose, glucose (DP = 1), LAM2 (DP = 2), LAM6 (DP = 6), and LAM9 (DP = 9), and mixture of UDP-glucose with glucose, LAM2, LAM6, or LAM9 as substrate, respectively. As shown in Fig. 3A, B, GFGLS2 showed a highest specific activity of 60.01 pmol min^−1^ μg^−1^ with UDP-glucose as substrate with V_max_ of 1.29 ± 0.04 µM min^−1^, kcat of 1.31 ± 0.04 min^−1^, and kcat/K_m_ of 0.019 ± 0.001 min^−1^ µM^−1^, while almost no activity was observed when using glucose or oligosaccharides with DPs of 2, 6, and 9 as substrate. Mixing the glucose or oligosaccharides as the acceptor with UDP–glucose as donor seems to have no significant effect on the activity of GFGLS2, which meant that the initiation of β-1, 3-glucan synthesis only requires the UDP–glucose as primer by self-priming without exogenously added other priming substrates.

The binding models of GFGLS2 with UDP–glucose, glucose, and oligosaccharides were further determined using differential scanning fluorescence (DSF). As shown in Fig. 3C, the melting temperatures (T_m_) of GFGLS2, and GFGLS2–UDP–glucose were 45.5 ± 0.2 °C and 45.0 ± 0.08 °C. The thermal shift value ΔT_m_ (0.5 ℃) was over twice that of the estimated standard deviation (0.2 °C) of GFGLS2, which indicated that a tight binding between GFGLS2 and UDP–glucose existed [16]. By contrast, all the ΔT_m_ values of GFGLS2 with the GFGLS2–glucose, GFGLS2–LAM6, and GFGLS2–LAM9 were less than twice that of the estimated standard deviation (0.2 °C) of GFGLS2, suggesting that GFGLS2 had no tight binding to those substrates, which confirmed the strict substrate specificity of GFGLS2 to UDP–glucose (Fig. 3D).

We also tested the β-1, 3-glucan synthase activities of two recombinant hydrophilic cytoplasmic domains rGFGLS2-classI and rGFGLS2-classIII. As shown in Fig. 3A, B, rGFGLS2-classI (aa 1–377) had no β-1, 3-glucan synthase activity in all tested substrates. The central hydrophilic cytoplasmic domain rGFGLS2-classIII (aa 649–1181) showed a similar catalytic features using UDP–glucose as priming substrate, but significant lower activity of 14.09 pmol min^−1^ μg^−1^, and V_max_ of 0.67 ± 0.04 µM min^−1^, kcat of 0.68 ± 0.04 min^−1^, and kcat/K_m_ of 0.003 ± 0.00027 min^−1^ µM^−1^ than those of partially-purified GFGLS2.

### Thermo-stability of GFGLS2 and rGFGLS2-classIII

**Fig. 4:**
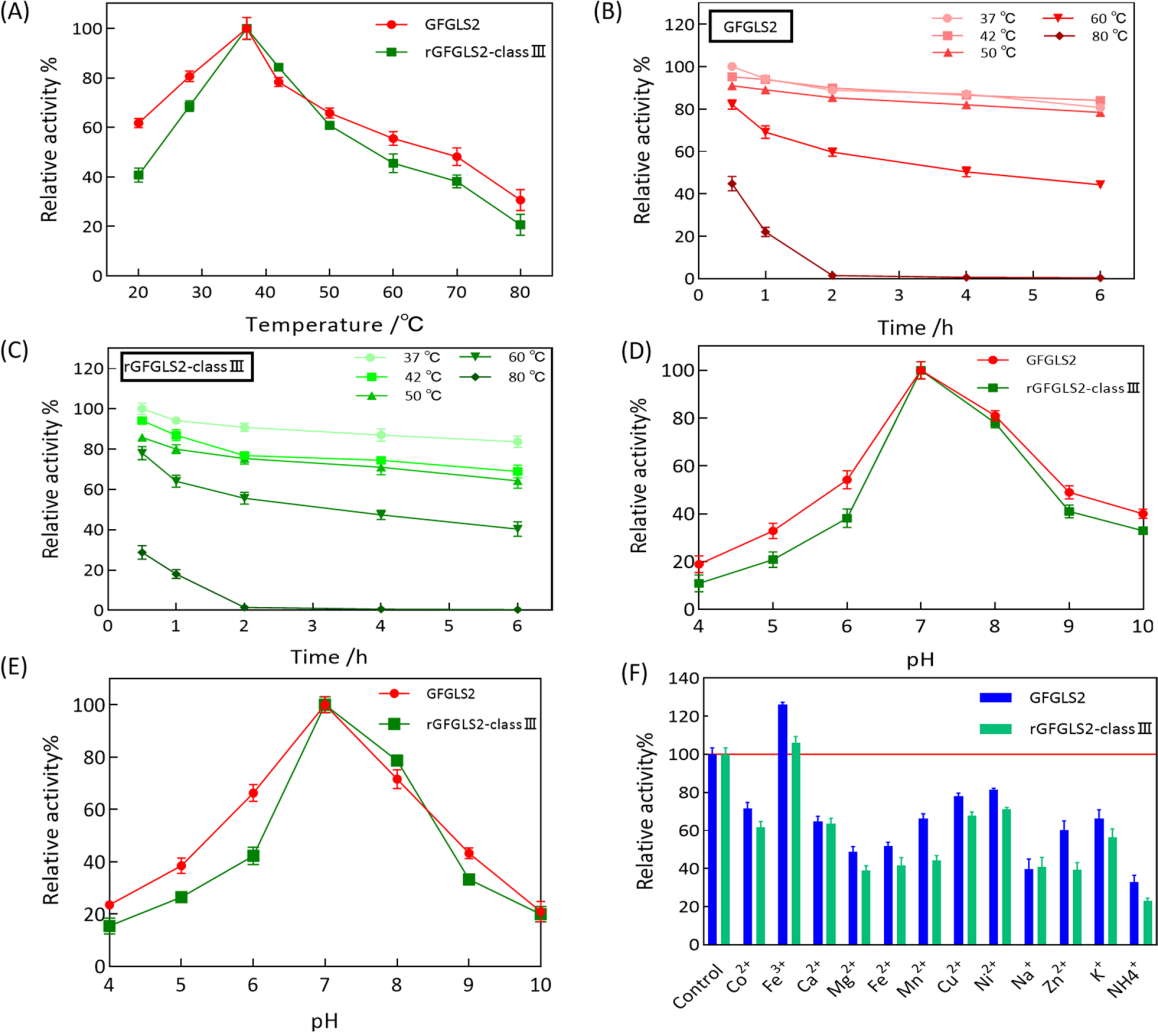
Optimal temperature for GFGLS2 and rGFGLS2-classIII activity (**A**). Thermal stability of GFGLS2 and rGFGLS2-classIII activity with UDP-Glu as substrate (**B**, **C**). Optimal pH for GFGLS2 and rGFGLS2-classIII activity (**D**). The pH stability for GFGLS2 and rGFGLS2-classIII activity (**E**). Effects of metal ions on the GFGLS2 and rGFGLS2-classIII activity at the concentration of 1.0 mM) (**F**)

Figure 4A–C presents effect of eight reaction temperatures ranging from 20 ℃ to 80 ℃ on activities of GFGLS2 and rGFGLS2-classIII. Their specific activities showed an increased trend to the maximum levels with an increase of temperature from 20 ℃ to 37 ℃, followed by a decline in activity to approximately 50% at 60 ℃. Hence, 37 ℃ may be regarded as the optimal temperature for GFGLS2 to catalyze the formation of β-1, 3-glucan chain.

GFGLS2 and rGFGLS2-classIII further were kept at temperatures ranging from 37 ℃ to 80 ℃ for 0.5–6 h. Maintaining at temperature of below 42 °C for less than 6 h kept their high residual activities of > 70%, while the increase of temperature to 60 °C significantly decreased to their relative activities to approximately 40%. No residual activity was detected after incubation at 80 °C for 2 h.

### pH-stability of GFGLS2 and rGFGLS2-classIII

As shown in Fig. 4D, the specific activities of GFGLS2 and rGFGLS2-classIII were highest at pH 7.0 and remarkably decreased approximately 40–80% at pHs from 4.0 to 6.0 and 9.0 to 10.0. GFGLS2 and rGFGLS2-classIII had stable activity at pH 7.0 after 1 h of storage at 37 °C, retained ~ 75% of residual activity at pH 8.0, and lost 80% of activity at pH 10.0 (Fig. 4E).

### Effect of metal ions

Figure 4F illustrates effect of metal ions including Co^2+^, Fe^3+^, Ca^2+^, Mg^2+^, Fe^2+^, Mn^2+^, Cu^2+^, Ni^2+^, Na^+^, Zn^2+^, K^+^, and NH_4_^+^ on GFGLS2 and rGFGLS2-classIII activity. Among them, only Fe^3+^ at 1.0 mM increased the GFGLS2 and rGFGLS2-classIII activities by approximately 25%, while others had a negative effect with the residual activities less than that of the control.

### Analysis of synthesized oligosaccharides

**Fig. 5:**
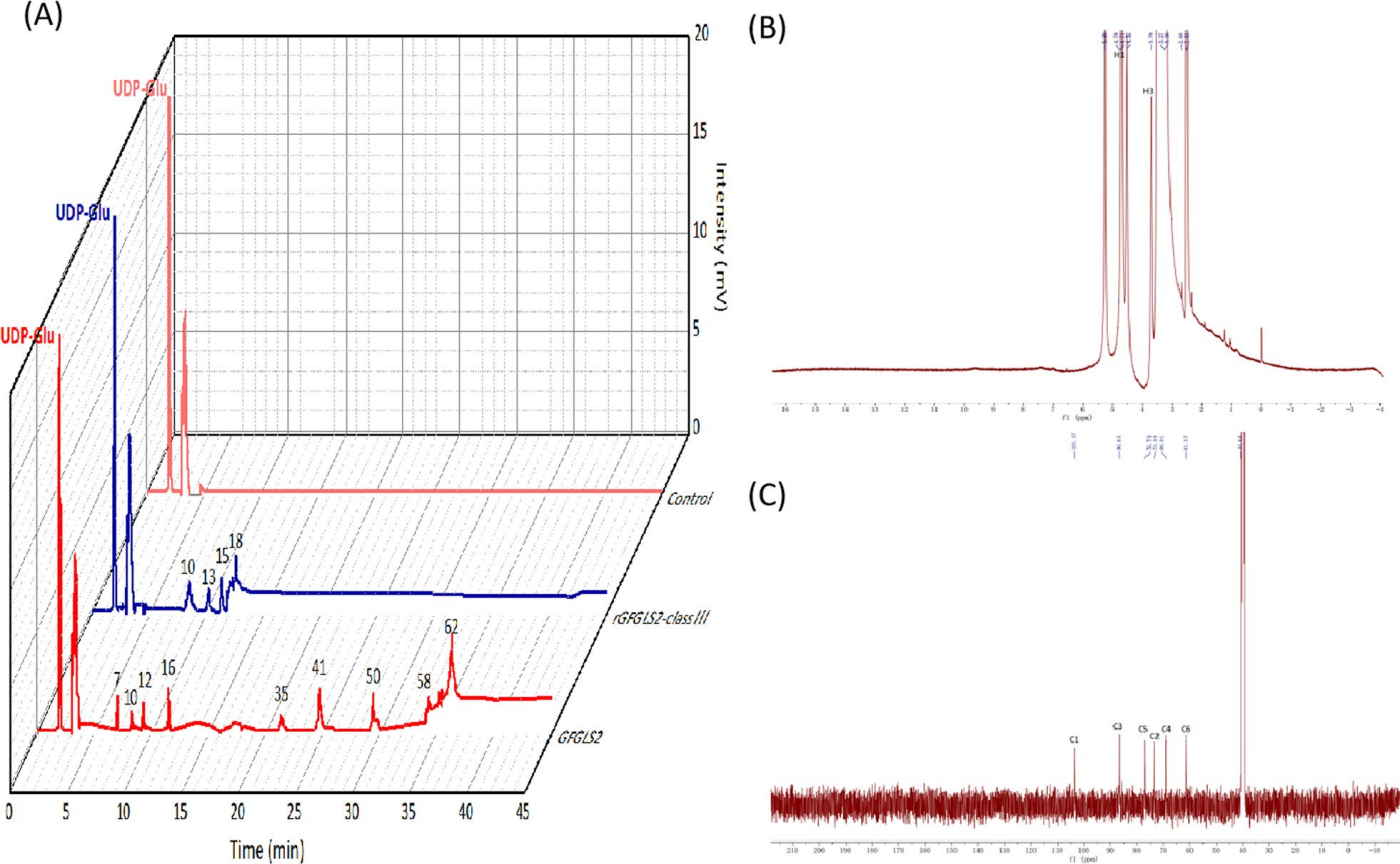
High Performance Liquid Chromatography (HPLC) chromatograms and ^1^H and ^13^C NMR spectroscopy of the synthesized products. **A** HPLC-ELSD chromatograms with DPs of the synthesized glucans of control (UDP-Glu standards, catalytic time of 0 h) and the 24 h catalyzed products by purified GFGLS2 and rGFGLS2-classIII. **B** NMR spectra on an ARX 600 MHz spectrometer in DMSO-d6 at 343.15 K ^1^H NMR spectrum, **C** ^13^C NMR spectrum

On the basis of our previously developed calibration curve of glucose, LAM2, LAM6, and LAM9 with their retention time on HPLC–ELSD [10] and the peaks appearing at the retention time from 7.01 to 36.17 min (Fig. 5A), DPs of GFGLS2 and rGFGLS2-classIII-catalyzed products using UDP-glucose as substrate were calculated to 7 ~ 62. In addition, the peak at 36.17 min occupied a high percentage of 75% among those at 7.01 to 36.17 min suggesting the synthesized glucan accumulated with DP of 62, which were in accordance with those synthesized by *S. cerevisiae* glucan synthase with the length of 60–80 mer [17, 18].

The ^1^H and ^13^C NMR spectroscopy were applied to verify the anomeric configuration and glycosidic linkage of the GFGLS2 synthesized oligoglucoses or glucans. As presented in Fig. 5B, synthesized glucans of GFGLS2 had a typical β-glycosidic configuration in ^1^H NMR spectrum with chemical shifts of δ 4.79 in the anomeric region, which confirmed the β-anomericity of the glucosyl units. The ^13^C NMR spectrum of synthesized glucans exhibited six main signals (Fig. 5C). The 103.57 ppm signal in the anomeric region was attributed to C1 in the β-glucosyl units, while the signals at 73.40, 68.91, 76.79 and 61.37 ppm were assigned to C2, C4, C5 and C6, respectively [19]. The correlation at 86.65/3.70 ppm was attributed to the substituted C3 and its hydrogen (C3/H3), which is consistent with the presence of 1, 3-glycosidic linkages [20]. Thus, the GFGLS2 synthesized-oligoglucoses or glucan could be conformed to contain the β-configuration and 1, 3-linked β-D-glucosyl units.

### Clustering and phylogeny analysis of β-1, 3-glucan synthase GFGLS2

**Fig. 6:**
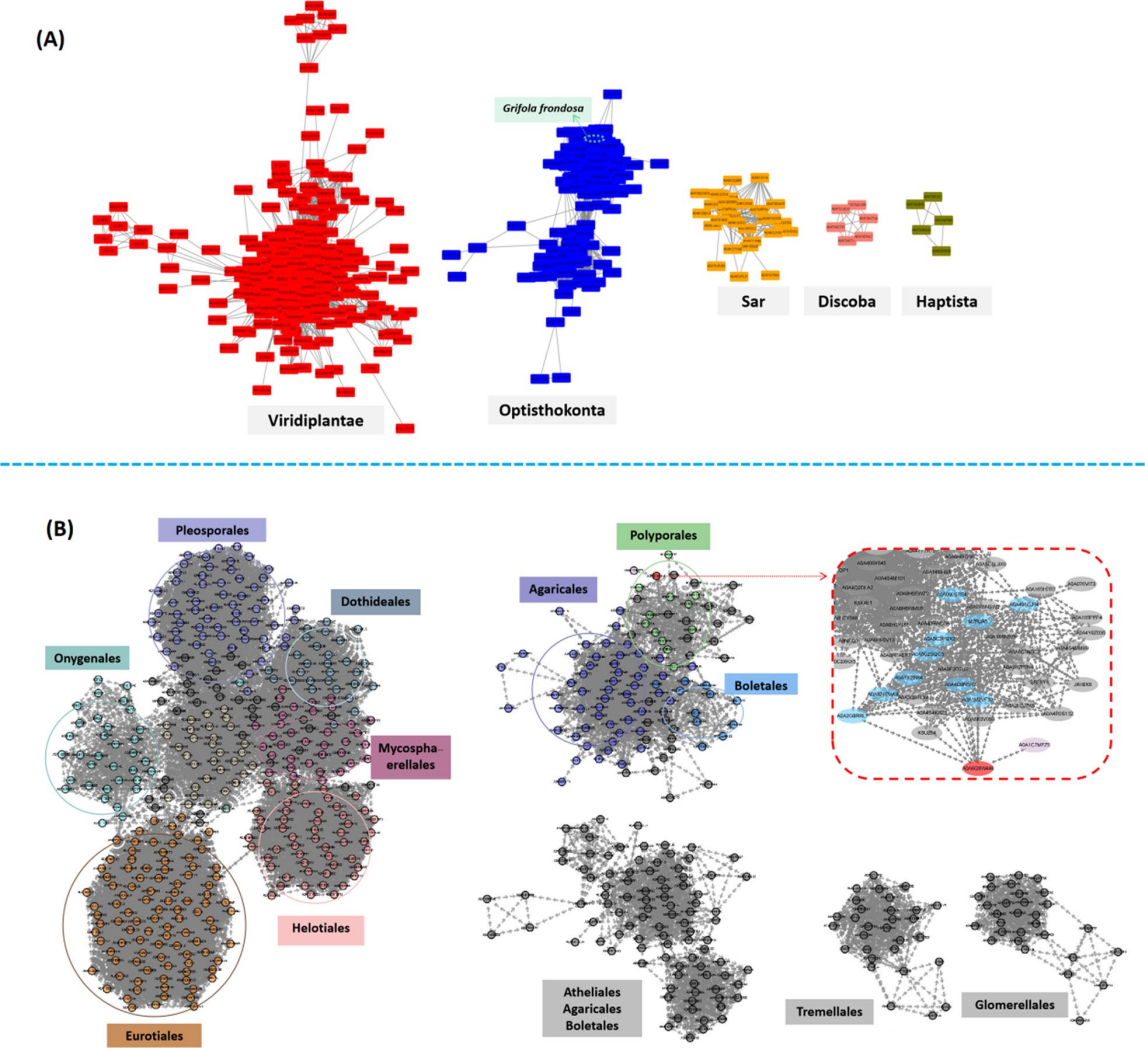
A sequence similarity network (SSN) for glucan synthase superfamily (PF02364) and full protein sequence of GFGLS2 were established by using Similarity Tool with EFI-EST to explore sequence-function space. The full network was downloaded and used to create a sequence similarity network using Cytoscape 3.9.1. **A** SSNs of glucan synthases superfamily (Pfam family 02364) in all kingdoms. **B** SSNs of full protein sequence of GFGLS2

To explore broadly insightful sequence similarity of glucan synthases superfamily (Pfam family 02364) in all kingdoms, sequence similarity networks (SSNs), based on all-*versus*-all pairwise local sequence alignments, was applied, computed and shown in Fig. 6A. Approximate 1079 sequences described with β-1, 3-glucan synthase activity were located in at least three main clusters of the kingdoms including *Optisthokonta*, *Viridiplantae*, *Haptista, Sar* and *Discoba*. 514 of β-1, 3-glucan synthases (some of them classified as Callose synthase) from plants and algae formatted the largest cluster with edges of 42,597, while 178 of β-1, 3-glucan synthases from *Saccharomyceta* and *Agaricomycetes,* located at a relative conserved cluster, respectively. Interestingly, from Fig. 6A, it could be observed that fungal β-1, 3-glucan synthases including GFGLS2 potentially shared a common ancestor with no relationship with those from plants or algae.

Full protein sequence of GFGLS2 was used as the query for a search of the UniProt, UniRef90, or UniRef50 database to generate a SSN for finding its closest homologues using the BLAST Retrieval Options. As shown in Fig. 6B, 761 annotated β-1, 3-glucan synthases belonging to *Eukaryota, Opisthokonta* were classified to over 10 clusters with the identity of > 85%. The largest cluster of 381 β-1, 3-glucan synthases was originated from lower fungi in the Orders of *Dothideales*, *Eurotiales, Helotiales*, *Mycosphaerellales, Onygenales* and *Pleosporales* in Phylum *Ascomycota* and Class *Saccharomyceta*. GFGLS2 was clustered to the second one composing of 106 β-1, 3-glucan synthases from edible/medicinal fungi in Orders of *Agaricales*, *Boletales* and *Polyporales* in Phylum *Basidiomycota* and Class *Agaricomycetes*. Interestingly, GFGLS2 had the direct relationship with other β-1, 3-glucan synthases in *Ganoderma sinense* ZZ0214-1, *Trametes coccinea* BRFM310, *Polyporus brumalis*, and *Trametes pubescens.*

### 3D model construction of GFGLS2 using AlphaFold 2

**Fig. 7:**
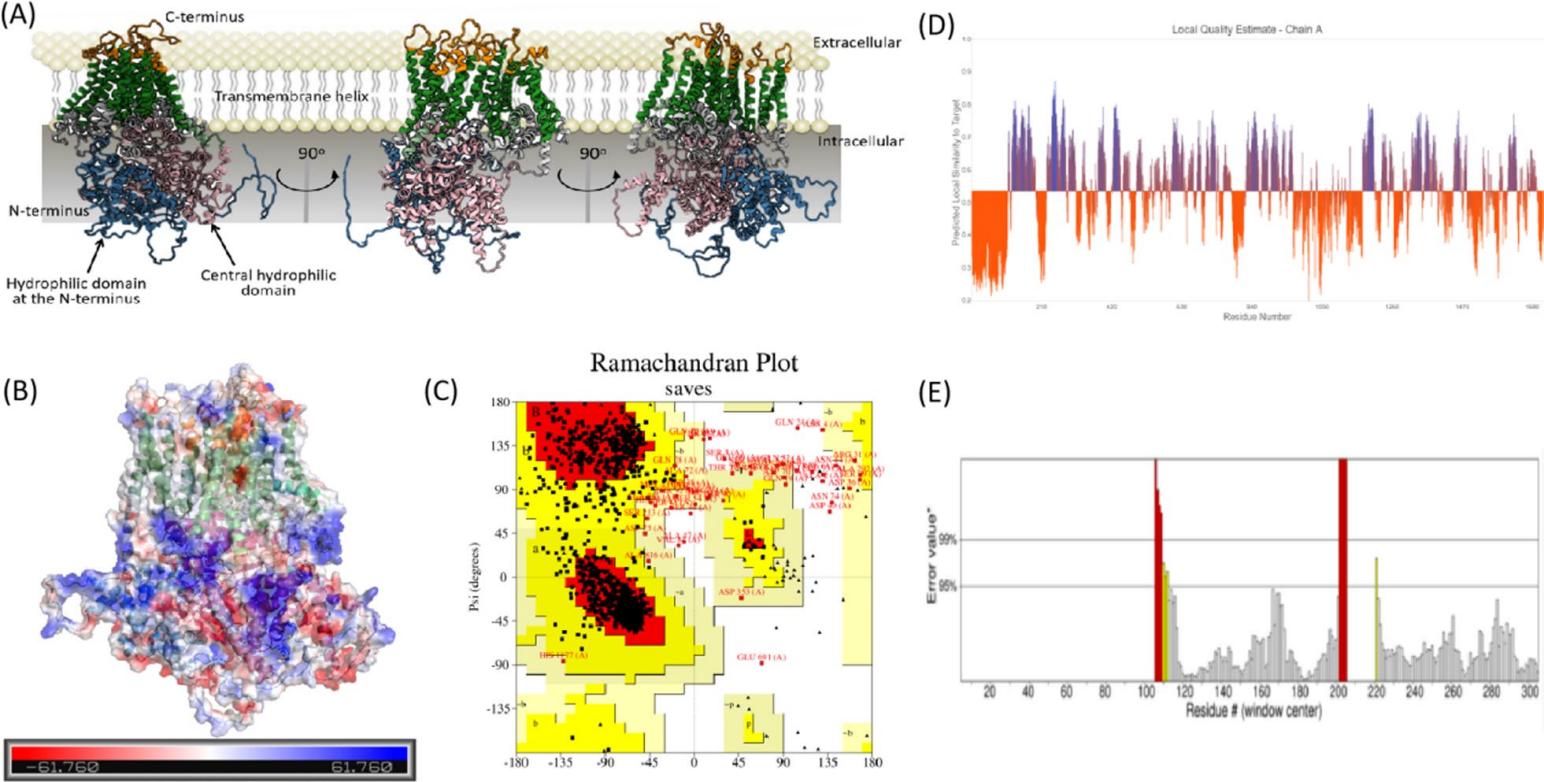
3D model of GFGLS2p constructed by the AlphaFold webserver (**A**). Overall charge distribution of GFGLS2 with the electrostatic potential difference from—61.760 to + 61.760 (**B**). The Ramachandran plot paragraph of GFGLS (**C**). The QMEANDisCo global score of GFGLS2 3D model evaluation (**D**). Overall quality of the GFGLS2 3D model evaluated by the ERRAT program (**E**)

The 3D structure of *G. frondosa* β-1, 3-glucan synthase GFGLS2p was predicted via an Alpha-Fold 2 webserver to format a PDB file to evaluate its reliability. The 3D structure of *G. frondosa* β-1, 3-glucan synthase GFGLS2p was visualized and analyzed using with PyMOL (Schrödinger, LLC) and Chimera X 1.4. As shown in Fig. 7A, GFGLS2 showed a sandwiched shape as extrinsic (orange label), transmembrane (dark green label), and periplasmic modules (blue for hydrophilic domain at the N-terminus, pink for the central hydrophilic domain, and gray for short hydrophilic loop connecting TMHs). TM8-14 formed a narrow channel for translocating the synthesized glucan chains to the cell wall space. The electrostatic potential map using coloring scheme displays the overall charge distribution of GFGLS2p (Fig. 7B) with an electrostatic potential difference from −61.760 to + 61.760. The TMHs domains in GFGLS2p showed the neutral potential (white), while the negative (red) and positive (blue) potentials were regionally distributed on the surfaces of extrinsic and periplasmic regions [21]. From Ramachandran plot analysis results shown in Fig. 7C, 87.8%, 9.0% and 2.2% of amino acids in GFGLS2 lay in the most favored region (Red), additional allowed regions (yellow) and generously allowed regions (light yellow), respectively. The QMEANDisCo global and ERRAT scores were 0.54 ± 0.05 and 91.56, respectively, which confirmed that the predicted model of GFGLS2 fit well within the range of a high quality (Fig. 7D, E).

### Insights into binding sites and interaction mechanism of GFGLS2 with UDP–glucose

**Fig. 8:**
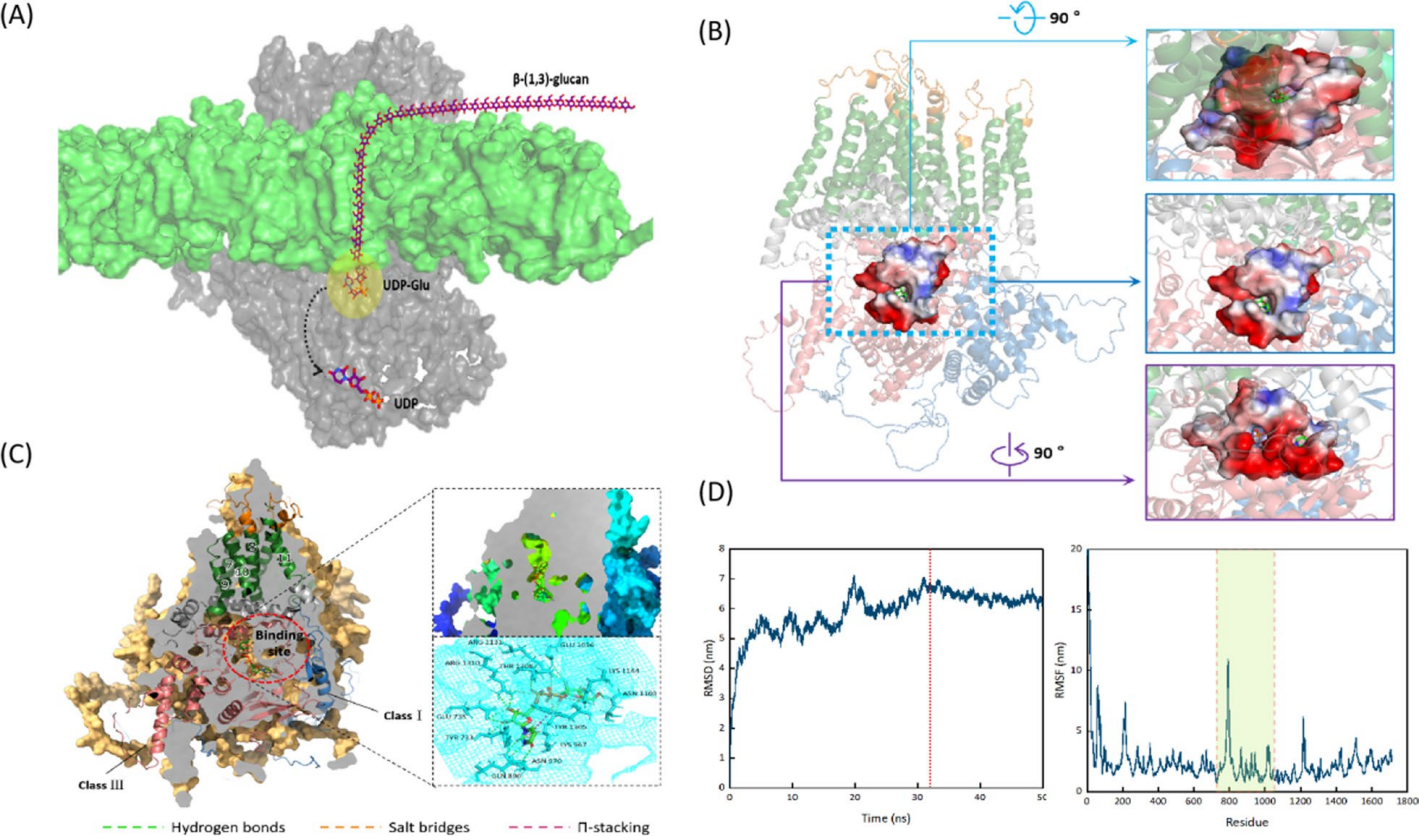
Schematic representation of substrate UDP-glucose interactions with active site residues of GFGLS2 calculated using the PyMOL program and Molecular dynamic simulations using GROMACS employed to confirm the binding affinity prediction of GFGLS2 and UDP-glucose. **A** Multi-modular structure of GFGLS2. GFGLS2 uses UDP-Glu to synthesize β-1, 3-glucan. **B** The potential binding pocket in GFGLS2. **C** A cutaway surface representation of GFGLS2 showing the binding sites with UDP-Glu and mesh representation of the binding of UDP-Glu and key residues with hydrogen bonds, salt bridges and π-stackingshown as green dotted line, orange dotted line, and pink dotted line. **D** Backbone RMSD of the complex and the ligand over the time scale of 50 ns and C-α RMSF profile of theGFGLS2-UDP-Glu complex

The binding sites and interaction mechanism of fungal β-1, 3-glucan synthases and substrate UDP–glucose are still not well discovered due to the lack of their 3D structures. On the basis of the developed 3D structure of using Alpha-Fold 2, the docking model of GFGLS2 with substrate UDP–glucose (CID: 8629) was constructed using AutoDock 4.2.6 software (Fig. 8A, B). A stable GFGLS2–UDP–glucose complex with the lowest binding energy of −5.98 kcal/mol was selected to analyze the potential active sites and interaction bonds. As shown in Fig. 8C, substrate UDP–glucose was packed by binding the residues including Tyr733, Glu735, Gln890, Lys967, Asn970, Asn1103, Arg1131, Lys1144, Tyr1305, Thr1308 and Arg1310 in the central hydrophilic domain (Class III) and the short hydrophilic loop connecting TM8–9, which proved that the recombinant GFGLS2-ClassIII showed a positive β-1, 3-glucan synthase activity. The O3, O5 and O6 moiety on glucose hydrogen-bound to Arg 1131 ^NH2^, Lys 1144 ^NZ^, and Asn 1103 ^OD1^, respectively. The α-phosphate (near the glucose moiety) interacted with Arg 1131 ^NH2^, Lys 967 ^NZ63^ and Lys 1144 ^NZ^ via salt bridges, while the β-phosphate (near the ribose ring) interacted with Arg 1310 ^CH^ via a salt bridge and Thr 1308 ^OG1^ and Tyr 1305 ^HH^ by hydrogen bonds. The O2’ and O3’ moiety on the ribose ring hydrogen-bound to Tyr 733 ^H^, Glu 735 ^OE2^, and Arg 1310 ^NH1^. The uracil ring bound to Tyr 1305 via π-stacking and Asn 970 ^ND2^ and Gln 890 ^OE1, NE2^ was bound by hydrogen bonds.

Molecular dynamic simulations using GROMACS were further employed to confirm the binding affinity prediction of GFGLS2 and UDP–glucose-based on molecular docking [22]. The root-mean-square deviation (RMSD) gives a measure of the average atomic displacement between two different conformations during the MD simulation. As presented in Fig. 8E, GFGLS2p-UDP-glucose complex displayed least deviation with average RMSD of ~ 6.4 nm, and reached to be stable after 32 ns, indicating the binding of GFGLS2p and UDP-glucose entered a stable status for catalytic reaction. The root-mean-square fluctuations (RMSFs) of amino acid residues (aa 732 to aa 1040) in the central hydrophilic domain (Class III) had significantly fluctuated, proved the its higher flexibility (Fig. 8D).

## Discussion

In general, fungal β-1, 3-glucan synthases are the transmembrane proteins with over 100 kDa molecular mass and 10 transmembrane domains, which hampered their extraction/purification or recombinant expression with high purity. Chhetri et al. presented a detailed protocol for preparation of partially purified β-1, 3-glucan synthase from *S. cerevisiae* using the product entrapment method with glucan synthase purity of 20–30% on SDS–PAGE [23]. Our previously results have also predicted the β-1, 3-glucan synthases GFGLS2p (Mw 195.2 kDa) and GFGLSp (Mw 203.66 kDa) in *G. frondosa* belonging to the integral membrane protein with two large membrane domains of 6 transmembrane helices (TMHs) at the N-terminus and 9 TMHs at the C-terminus [5]. Herein, we attempted to extract and partially purify the β-1, 3-glucan synthase from the cultured mycelia of *G. frondosa* CGMCC 5.626 using modified product entrapment method followed with Superdex 200 Increase 10/300 GL chromatography. The trypsin digestion and Mascot search analysis showed that the synthase complex (GSC) in *G. frondosa* should be composed of β-1, 3-glucan synthase GFGLS2 and RhoA. Similarly, Ouyang et al. also identified β-1, 3-glucan synthase catalytic subunit FksP (gi|70992539) using 2D LC–MS/MS with Mws of 219.8 kDa and GPI-anchored membrane proteins of *A. fumigatus* [24]. In addition, RhoA is also proved to participate in maintaining cell integrity, establishing polarity and synthesizing cell wall during the spore germination process of *Phycomyces blakesleeanus* and *A. nidulans* [25, 26].

Biosynthesis of β-1, 3-glucan chain requires the glycosyltransferases (GTs) transferring the glucose moiety to the existing sugar chains. According to the carbohydrate-active enzyme database CAZy (http://www.cazy.org/), glycosyltransferases are categorized into three types of GT-A, GT-B, and GT-C according to their catalytic domains, and β-1, 3-glucan synthase should be classified as GT-C type since they belong to the membrane-bound proteins containing the transmembrane helices. Chhetri et al. deduced that β-1, 3-glucan synthase uses the glucose moiety hydrolyzed from the first UDP–glucose as priming substrate [23]. GFGLS2 showed a highest specific activity with UDP–glucose as substrate. Almost no activity was observed when using glucose or oligosaccharides as substrate. Mixing the glucose or oligosaccharides as the acceptor with UDP–glucose as donor seems to have no significant effect on the activity of GFGLS2 (Fig. 3A, B). Similar results were also found by Fu et al., that *C. militaris* β-1, 3-glucan synthase CMGLS had a strict substrate specificity to UDP–glucose with the highest specific activity with no need of priming acceptor to polymerize glucan chains [10]. From DSF analysis results, ΔT_m_ confirmed the strict substrate specificity of GFGLS2 to UDP-glucose and central hydrophilic cytoplasmic domain rGFGLS2-classIII mainly responsible for the catalytic activity of β-1, 3-glucan synthase GFGLS2 (Fig. 3C, D). Similarly, Tomazett et al. also observed the recombinant hydrophilic domain (*Pb*Fks1pc, 57 kDa, aa 796–1331) of β-1, 3-glucan synthase (*PbFKS1*) from *Paracoccidioides brasiliensis* Pb01 could incorporate 1.27 × 10^–8^ nM of glucose to β-1, 3-glucan within 1-h reaction [27].

As shown in Fig. 4A–C, 37 ℃ may be regarded as the optimal temperature for GFGLS2 and rGFGLS2-classIII to catalyze the formation of β-1, 3-glucan chain. However, β-glucan synthases from *Pyricularia oryzae* and *Candida albicans* showed the highest relative activities at 20 °C and 30 °C, respectively [28, 29]. The specific activities of GFGLS2 and rGFGLS2-classIII were highest at pH 7.0 and had stable activity at pH 7.0 after 1 h of storage at 37 °C (Fig. 4D, E). Févre and Dumas found β-glucan synthetases from *Saprolegnia monoica* had the optimum pH of 5.8, while Wang and Bartnicki-Garcia observed the optimal pH for GLS of *Phytophthora palmivora* was 7.8 [30, 31]. Only metal ion Fe^3+^ increased the GFGLS2 and rGFGLS2-classIII activities evidently (Fig. 4F). However, San-Blas found that Ca^2+^ was the best stimulator for *P. brasiliensis* GLS [32], while metal ions Mg^2+^ and Ca^2+^, GTP, EDTA and EGTA displayed no effect on β-1, 3-glucan synthase activity of *Phytophthora sojae* and both Mn^2+^ and Fe^2+^ inhibited enzyme activity to about 70% at 10 mM [33]. The HPLC–ELSD and NMR spectroscopy results confirmed GFGLS2 synthesized-oligoglucoses or glucans with DPs of < 62 containing β-configuration and 1, 3-linked β-D-glucosyl units.

Previously, we have revealed that the deduced GFGLS2p shared 72.6% similarity with GFGLSp and high homology of 93.10% and 91.24% with those in edible fungi *S. crispa* and *Steccherinum ochraceum*, respectively, using multiple sequence alignment (MSA) and subsequent phylogenetic tree estimation [5]. Using sequence similarity networks (SSNs) analysis, GFGLS2 potentially shared a common ancestor with other fungal β-1, 3-glucan synthases and the direct relationship with GLSs in *Ganoderma sinense* ZZ0214-1, *Trametes coccinea* BRFM310, *Polyporus brumalis* and *Trametes pubescens*, while having no relationship with those from plants or algae.

The fine architectures of enzymes are the basis to understand their catalytic processes. However, the β-1, 3-glucan synthases had the extremely complex structures with multi-transmembrane helices, which bottlenecked to determine their crystal structures for explain the detailed β-1, 3-glucan synthesis process. Artificial intelligence such as AlphaFold 2 has proved as the powerful tools and predicted over 350, 000 protein models from various species [34]. The 3D structure of *G. frondosa* β-1, 3-glucan synthase GFGLS2p predicted using Alpha-Fold 2 showed a typical sandwiched appearance as extrinsic, transmembrane and periplasmic modules. A narrow channel formed by TM8-14 should present the function to translocate the synthesized glucans to the cell wall space. Through analysis into binding sites and interaction mechanism of GFGLS2 with UDP–glucose (Fig. 8C), Arg 1131 could be regarded as a key residue having a direct interaction with moieties of glucose and UDP, which was different from previous results of CMGLS from *C. militaris* with Arg 1436 binding the moieties of glucose, phosphate and ribose ring on UDP–glucose [10].

## Conclusions

In the present study, the membrane-bound β-1, 3-glucan synthase GFGLS2 was purified from cultured mycelia of *G. frondosa* with a specific activity of 60.01 pmol min^−1^ μg^−1^ for the first time. GFGLS2 showed a strict specificity to UDP–glucose with a V_max_ value of 1.29 ± 0.04 µM min^−1^ at pH 7.0. SSNs analysis proved that GFGLS2 has a close relationship with others in *Ganoderma sinense*, *Trametes coccinea*, *Polyporus brumalis*, and *Trametes pubescens*. A fine 3D structure of GFGLS2 was constructed for the first time to our knowledge using AlphaFold 2.0. Molecular docking and molecular dynamics simulations proved that substrate UDP-glucose was potentially bound to at least 11 residues via hydrogen bonds, π-stacking and salt bridges, and Arg 1131 could be regarded as a key residue having a direct interaction with moieties of glucose and UDP. These findings would provide a reference for elucidating the potential catalytic mechanism of β-1, 3-glucan synthases to synthesize the glucan chain in edible fungi.

## Methods

### Strains and media

*G. frondosa* CGMCC 5.626 used in the present study is kept on potato dextrose agar (PDA) slants at 4 °C in our laboratory [5]. A stock culture of *G. frondosa* on PDA plants was activated on CYM plates (maltose 10 g/L, glucose 20 g/L, yeast extract 2 g/L, tryptone 2 g/L, MgSO_4_·7H_2_O 0.5 g/L, KH_2_PO_4_ 0.5 g/L and 2.0% agar) incubated at 28 °C for 7 days. *G. frondosa* seed culture on CYM liquid medium was prepared by inoculating five pieces (about 5 mm) of the activated culture grown at 28 °C on a rotary shaker incubator at 150 rpm for 7 days. The chemical materials, unless otherwise specified, were of analytical grade and purchased from Sinopharm Chemical Reagent Co., Ltd. (Shanghai, China). The fermented broth was centrifuged at 5000*g* for 20 min at 4 °C to collect the mycelia for purification of the β-1, 3-glucan synthases.

### Extracting and purifying membrane-bound β-1, 3-glucan synthases

Membrane-bound β-1, 3-glucan synthases of *G. frondosa* was prepared according to Chhetri et al.’s method with some modifications [10, 23]. Briefly, the cultured mycelia of *G. frondosa* were suspended in an ice-cold breaking buffer containing 0.5 M sodium chloride, 10 mM EDTA stock (pH 8.0), and 1 mM PMSF for 10-min ultrasonic extraction. The membrane pellets was collected after ultracentrifugation at 100000*g* for 1 h at 4 °C of the extract supernatant, and resuspended in a membrane buffer containing 50 mM Tris (pH 8.0), 33% (v/v) glycerol, and 10 mM EDTA followed by adding 27.5 μM GTPγS, 6.88 mM DTT, 192 mM NaCl, and detergents including 0.688% (w/v) CHAPS and 0.138% (w/v) CHS. The entrapped β-1, 3-glucan synthases were collected from 20-min reaction suspension after ultracentrifugation at 100, 000 g for 30 min at 4 °C. Five millimoles of UDP–glucose and KF were added into the supernatant for glucan synthesis at 37 °C for 12 h. After the 30 min ice bath, the β-1, 3-glucan synthases were collected by centrifugation at 8000*g* for 10 min at 4 °C; washed with a washing buffer containing 5 mM UDP-glucose, 1 mM DTT, and 4 μM GTPγS three times; and dissolved in an extraction buffer (0.4% CHAPS, 0.08% CHS, 1 mM DTT, and 4 μM GTPγS in the membrane buffer) to obtain the crude β-1, 3-glucan synthases of *G. frondosa*. The crude β-1, 3-glucan synthase sample was applied onto Superdex 200 Increase 10/300 GL (300 × 10 mm, i.d.) (Cytiva, Global Life Sciences Solutions, MA, USA) using the AKTA-purifier chromatography system (GE Healthcare Life Sciences, Uppsala, Sweden) at room temperature (25  ℃) and eluted with washing buffer containing 50 mM Tris–HCl, 10 mM EDTA, 6.8 mM DTT and 1.92 mM NaCl for further purification at a flow rate of 0.50 mL/min.

### Identification of purified membrane-bound β-1, 3-glucan synthases

The purity of purified membrane-bound β-1, 3-glucan synthases and its native molecular masses were estimated using sodium dodecyl sulfate–polyacrylamide gel electrophoresis (SDS–PAGE) after staining with a commercial Silver Staining Kit (Protein Stains K, Sangon Co., Ltd, Shanghai China) [35]. The molecular mass was calculated by comparison to a premixed protein marker with the molecular masses ranging from 10 to 245 kDa (Takara Bio Inc., Dalian, China).

The bands on SDS–PAGE gel were excised, destained using 50% ACN-50% 50 mM NH_4_HCO_3_ solution, dehydrated using 100 μL 100% ACN solution, and digested at 37 ℃ 16 h using 15 ng/μL trypsin, respectively. The trypsin hydrolysates were analyzed using a Q Exactive™ Hybrid Quadrupole-Orbitrap™ mass spectrometer (Thermo Fisher Scientific, Waltham, MA, USA) coupled with an Easy-nLC 1200 capillary high performance liquid chromatography (Thermo Fisher Scientific, Waltham, MA, USA) to predict its peptide masses and MS/MS spectra. Protein identification was performed by searching extracted peak lists against the NCBInr database (species restricted to *G. frondosa*) using the Mascot (Matrix Science, London, UK) search engine (MS/MS ion search, 1 miscleavage, precursor tolerance: 100 ppm, MS/MS tolerance: 0.5 Da) (*P* < 0.05).

### Recombination of hydrophilic cytoplasmic domains of GFGLS2

According to the predicted topology of GFGLS2p structure, two hydrophilic cytoplasmic domains (class I: aa 1–377 and class III: aa 649–1181) were heterologously expressed to check their potential β-1, 3-glucan synthase activity. The gene sequences of *gfgls2classI* and *gfgls2classIII* were codon-optimized, synthesized by GenScript (Nanjing, China) and then inserted into the vector pET-30a ( +) between restriction sites *NdeI* and *HindIII*, respectively (Table 1). The constructed plasmids pET-30a ( +)-*gfgls2classI* and pET-30a ( +)-*gfgls2classIII* were verified by *NdeI* and *HindIII* restriction digestions, sequenced and transformed into *Escherichia coli* BL21(DE3) to construct the expression strains *E. coli* BL21(DE3)/pET-30a ( +)-*gfgls2classI* and *E. coli* BL21(DE3)/pET-30a ( +)-*gfgls2classIII*.

**Table 1:** Primers used for cloning gDNA sequences of GFGLS2, and CDS sequences of gfgls2classI and gfgls2classIII

Primer	Sequence (5’ to 3’)	Description
GFGLS2-gDNA-F	ATGGCTTCCAATCCGGGCTATG	cloning of gDNA sequence of GFGLS2 from *G. frondosa* genome
GFGLS2-gDNA-R	TTAGATATTTTGGCAAACGGT	
gfgls2classI- F-NdeI	GGAATTCCATATGATGGCTTCAAATCCAGGTTA	Cloning the CDS sequence of gfgls2classI to pET-30a( +)
gfgls2classI- R-HindIII	CCCAAGCTTAGCTGAACGCTTTTCAAAAA	
gfgls2classIII- F-NdeI	GGAATTCCATATGTGGAAAGAGGTTTATACGCG	Cloning the CDS sequence of gfgls2classIII to pET-30a( +)
gfgls2classIII- R-HindIII	CCCAAGCTTTATGAAAACCGGGATGACCA	

Note: The sequences underlined are the restriction sites for NdeI and HindIII, respectively

*E. coli* cells were grown in LB medium supplemented with kanamycin (50 μg/mL) at 37 °C until the optical density at 600 nm reached 0.6–0.8. Protein expression was induced by adding IPTG (Sangon Biotech, Shanghai, China) to a final concentration of 0.5 mM for inducting protein expression at 16 h at 15 °C and 37 °C at 4 h with 200 rpm, respectively. The cell pellets were harvested by centrifugation at 8000*g* and 4 °C, resuspended in 100 mM phosphate-buffered saline (pH 7.4) and homogenized to obtain cell lysate supernatants via centrifugation at 4 °C and 13000*g* for 10 min.

The recombinant GFGLS2-classI and GFGLS2-classIII (rGFGLS2-classI and rGFGLS2-classIII) were extracted using Inclusion Body Protein Solubilization and Refolding Kit (Coolaber, Beijing, China) and purified on Ni–NTA-Sefinose™ column (Sangon Biotech, Shanghai, China) using buffer (0.5 M NaCl, 20 mM Tris, 500 mM imidazole, pH 8.0) at a flow rate of 0.6 mL/min. The homogeneity of purified GFGLS2-classI and GFGLS2-classIII was assessed via SDS-PAGE. Protein concentration of the purified protein was determined using a bicinchoninic assay kit (Sangon Biotech).

### Substrate specificity of GFGLS2, rGFGLS2-classI and rGFGLS2-classIII

As mentioned in previous studies [10, 36], the Glycosyltransferase Activity Kit (R&D System, Minneapolis, MN, USA) was used to determine the β-1, 3-glucan synthase activity of GFGLS2, rGFGLS2-classI and rGFGLS2-classIII by measuring the levels of inorganic phosphate released from UDP after the glycosylation reaction with UDP-glucose (Sigma-Aldrich, St. Louis, USA) as substrate [36]. The specific activity of β-1, 3-glucan synthase was defined as the amount of 1 pmol of phosphate released per microgram of protein (μg) per unit time (min) (pmol min^−1^ μg^−1^).

The substrate specificities of GFGLS2, rGFGLS2-classI and rGFGLS2-classIII were determined at 37 °C and pH 7.0 for 24 h using the reaction mixture containing 10 mM Tris–HCl (pH 7.0), 2.5 μg of the purified protein, and 5 mM of various substrates including UDP-glucose, glucose, laminaribiose (LAM2), laminarihexaose (LAM6), laminarinonaose (LAM9) (Megazyme, Wicklow, Ireland), and mixture of UDP-glucose with glucose, LAM2, LAM6, or LAM9, respectively. The Malachite reagents A and B were added to each well, and then the OD_620nm_ values representing the levels of inorganic phosphate released from UDP were determined using a plate reader (Tecan Infinite M200 PRO, Männedorf, Switzerland) to calculate their specific activity of β-1, 3-glucan synthase.

### Enzymatic characterization of GFGLS2 and rGFGLS2-classIII

The optimal temperatures for GFGLS2 and rGFGLS2-classIII were determined with the reaction temperatures ranging from 20 to 80 °C at pH 7.0 for 24 h. Their thermostability was analyzed by keeping GFGLS2 and rGFGLS2-classIII at temperatures ranging from 20 to 80 °C at pH 7.0 for 0.5, 1, 2, 4, and 6 h to determine their residual activity. The optimum pH and pH stability were determined by measuring glucan synthase activity at 37 °C and pH 4.0–10.0 using 50 mM of the citrate buffer (pH 4.0–6.0), Tris–HCl buffer (pH 7.0–9.0), and glycine–NaOH buffer (pH 10.0) and stored at 37 °C and pH 4.0–10.0 for 60 min. Kinetic parameters (K_m_, kcat, kcat/K_m_) of the Michaelis–Menten equation for GFGLS2 and rGFGLS2-classIII were determined by measuring initial rates of the reaction with different concentrations of UDP–glucose (0–500 μM) under the optimal temperature and pH. The kinetic constants were obtained by fitting the curve of the Michaelis − Menten equation using GraphPad Prism 9.0 (GraphPad Software, CA, USA). The effect of ions Co^2+^, Fe^3+^, Ca^2+^, Mg^2+^, Fe^2+^, Mn^2+^, Cu^2+^, Ni^2+^, Na^+^, Zn^2+^, K^+^, and NH_4_^+^ on the activity of GFGLS2 and rGFGLS2-classIII was tested by incubation with 1 mM final concentration at pH 7.0 and 37 °C for 24 h.

### Verification of reaction products

The 24-h reactions of purified GFGLS2 with UDP–glucose was terminated by adding 200 μL ice-cold methanol. The reaction mixture was loaded onto an LC-20AD HPLC System (Shimadzu Corporation, Tokyo, Japan) coupled with an Asahipak NH2P-50 4E column (250 × 4.6 mm; Shodex China Co., Ltd., Shanghai, China) with a Schambeck ZAM 4000 evaporative light scattering detector, and eluted with acetonitrile/water 57/43 at 30 ℃ with a flow rate of 1.0 mL/min. The degree of polymerizations (DPs) of reaction products were calculated via the established calibration curve as previously described [10].

The reaction product was dissolved in 200 μL dimethyl sulfoxide-d6 (DMSO-d_6_, 99.9% isotropic purity, Sigma-Aldrich) for ^13^C and ^1^H NMR analysis at the frequency of 400 and 101 MHz on an ARX 600 MHz spectrometer (Bruker, Karlsruhe, Germany) at 343.15 K, respectively. The ^1^H and ^13^C chemical shifts were calibrated using the methyl resonances of residual DMSO-d_6_ at 2.52 ppm for ^1^H and 39.52 ppm for ^13^C [37].

### Substrates–GFGLS2 binding analysis using differential scanning fluorescence

GFGLS2p was incubated with 50 μM various substrates including UDP–glucose, glucose, LAM2, LAM6, LAM9 (Megazyme, Wicklow, Ireland), and mixture of UDP-glucose with glucose, LAM2, LAM6, or LAM9 in 10 μL Prometheus series capillaries. The samples were heated in Monolith NT.48 from 20 to 90 °C in 1.0 °C steps. The data were processed with PR TermControl software and presented with Origin 2021.

### Sequence similarity network and 3D structure prediction of GFGLS2

On the basis of the our previously annotated sequence of the putative *G. frondosa* β-1, 3-glucan synthase gene *GFGLS2* (GenBank: MK477285.1) [5], the protein sequence of GFGLS2p was aligned with NCBI Protein BLAST program choosing the nonredundant protein sequence database (https://www.ncbi.nlm.nih.gov/) and its homology and phylogenetic relationship was analyzed with tools of MEGA 64 software. Sequence similarity networks (SSNs) for glucan synthase superfamily (Option B, PF02364) and full protein sequence of GFGLS2 (Option A) were established using Similarity Tool with EFI–EST (https://efi.igb.illinois.edu/efi-est/) to explore sequence–function space [38]. A minimum edge threshold for the SSN was set as an alignment score of 118. The full network was downloaded and used to create a sequence similarity network using Cytoscape 3.9.1 [39].

The full sequence of *G. frondosa* β-1, 3-glucan synthase GFGLS2p (GenBank: QIR82081.1, 1713 amino acid residues) was subjected to an Alpha-Fold webserver (https://cryonet.ai/af2, provided by Prof. Qiangfeng Cliff Zhang’s Lab in Tsinghua University) to obtain the PDB file for predicting its 3D structure. The model accuracy or the model confidence was further checked using SAVESv6.0 analysis (https://saves.mbi.ucla.edu/) by a Ramachandran plot and ERRAT-score. Structures were visualized using Chimera X.

### Molecular docking and active site analysis of GFGLS2

The substrate UDP–glucose (CID: 8629, obtained from PubChem https://pubchem.ncbi.nlm.nih.gov/) was docked in the 3D structure of GFGLS2 using the AutoDock 4.2.6 software and MGLTools. The potential binding pockets were detected by the DoGSiteScorer webserver (https://proteins.plus/), and grids [centers (x, y, z) = (−3.51, 0.799, 0.204), size (X, Y, Z) = (94, 126, 114)] were used for docking the acceptors with 0.464 Å spacing. The simulation output results were sorted based on binding energy (kcal/mol). All docking results were visually inspected in Protein–Ligand Interaction Profiler webserver (https://plip-tool.biotec.tu-dresden.de/plip-web/plip/index) and Pymol software [40].

### Molecular dynamics (MD) simulations analysis molecular dynamics simulations

The docked complex structure of GFGLS2 and the substrate UDP–glucose was used as the starting structure of molecular dynamics simulations using GROMACS version 4.0 and OPLS-AA force field [41]. GFGLS2 was placed in the center of a cubic box consisting of SPC216 water molecules, with periodic boundary conditions. After neutralization the redundant charges, the energy of the system was minimized with the steepest descents method to obtain a reasonable geometrical structure. To maintain the simulated systems at a constant temperature and pressure, 100 ps NVT and 100 ps NPT ensembles were used to obtain the stable environment parameters. After completion of equilibration, 10 ns long MD simulations were performed with a step time of 2 fs at a constant temperature of 300 K and a constant pressure of 1 bar pressure, respectively. The root mean square fluctuation of residues (RMSF) and root mean square deviation from the initial structure (RMSD) were calculated using the corresponding built-in packages available in GROMACS. The dynamics trajectories were analyzed using VMD software, and Grace 5.1.25.

### Statistical analysis

The data generated was average of three independent experiments. The data were expressed as means ± standard deviations (*n* = 3). Statistical comparisons were made by one-way analysis of variance (ANOVA), followed by Duncan's multiple-comparison test. Differences were considered significant when the *P*–values were < 0.05.

## Data Availability

The authors declare that the data supporting the findings of this study are available within the paper and its Supplementary Information files. Should any raw data files be needed in another format they are available from the corresponding author upon reasonable request.
